# 5-(2-Methyl-5-nitro­phen­yl)-1*H*-tetra­zole

**DOI:** 10.1107/S1600536808029176

**Published:** 2008-09-17

**Authors:** Jing Dai, Wei Dai

**Affiliations:** a Ordered Matter Science Research Center, College of Chemistry and Chemical Engineering, Southeast University, Nanjing 210096, People’s Republic of China

## Abstract

In the title compound, C_8_H_7_N_5_O_2_, the benzene ring makes a dihedral angle of 45.7 (2)° with the tetra­zole ring. In the crystal structure, the mol­ecules are linked into a chain running along the *a* axis by N—H⋯N hydrogen bonds, and the chains are linked through π–π inter­actions between the tetra­zole rings [centroid–centroid distance = 3.450 (2) Å].

## Related literature

For the use of tetra­zole derivatives in coordination chemisty, see: Arp *et al.* (2000 ▶); Dai & Fu (2008 ▶); Wang *et al.* (2005 ▶); Xiong *et al.* (2002 ▶).
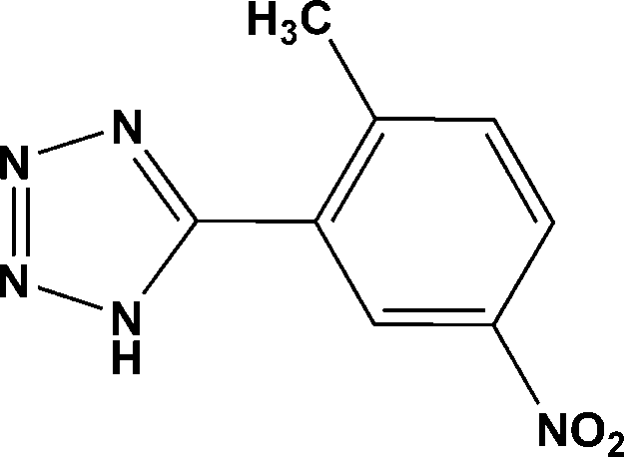


## Experimental

### 

#### Crystal data

C_8_H_7_N_5_O_2_
*M* *_r_* = 205.19
Monoclinic, 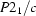

*a* = 4.9057 (10) Å
*b* = 16.938 (3) Å
*c* = 11.463 (2) Å
β = 98.65 (3)°
*V* = 941.7 (3) Å^3^
*Z* = 4
Mo *K*α radiation
μ = 0.11 mm^−1^
*T* = 298 (2) K
0.25 × 0.18 × 0.15 mm

#### Data collection

Rigaku Mercury2 diffractometer
Absorption correction: multi-scan (*CrystalClear*, Rigaku, 2005 ▶) *T*_min_ = 0.971, *T*_max_ = 0.977
9281 measured reflections
2085 independent reflections
1434 reflections with *I* > 2σ(*I*)
*R*_int_ = 0.056

#### Refinement

*R*[*F* ^2^ > 2σ(*F* ^2^)] = 0.082
*wR*(*F* ^2^) = 0.203
*S* = 1.13
2085 reflections
137 parameters
H-atom parameters constrained
Δρ_max_ = 0.30 e Å^−3^
Δρ_min_ = −0.29 e Å^−3^

### 

Data collection: *CrystalClear* (Rigaku, 2005 ▶); cell refinement: *CrystalClear*; data reduction: *CrystalClear*; program(s) used to solve structure: *SHELXS97* (Sheldrick, 2008 ▶); program(s) used to refine structure: *SHELXL97* (Sheldrick, 2008 ▶); molecular graphics: *SHELXTL* (Sheldrick, 2008 ▶); software used to prepare material for publication: *SHELXTL*.

## Supplementary Material

Crystal structure: contains datablocks I, New_Global_Publ_Block. DOI: 10.1107/S1600536808029176/ci2669sup1.cif

Structure factors: contains datablocks I. DOI: 10.1107/S1600536808029176/ci2669Isup2.hkl

Additional supplementary materials:  crystallographic information; 3D view; checkCIF report

## Figures and Tables

**Table 1:** Hydrogen-bond geometry (Å, °)

*D*—H⋯*A*	*D*—H	H⋯*A*	*D*⋯*A*	*D*—H⋯*A*
N1—H1⋯N4^i^	0.86	1.98	2.775 (4)	154

Symmetry code: (i) 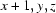.

**Fig. 1.**
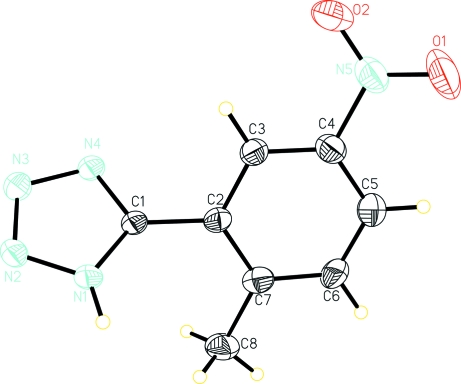
The molecular structure of the title compound, showing the atomic numbering scheme. Displacement ellipsoids are drawn at the 30% probability level.

**Fig. 2.**
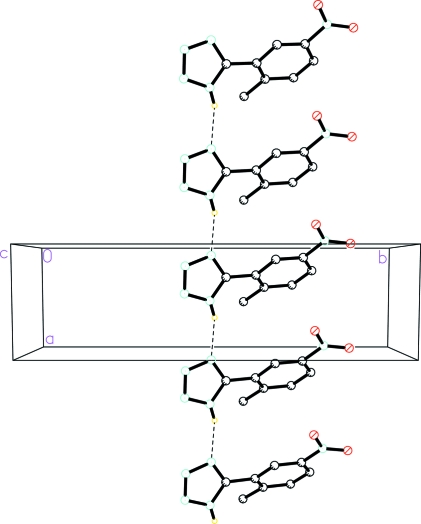
Part of the crystal packing of the title compound, showing a hydrogen- bonded (dashed lines) chain running along the a axis. H atoms not involved in hydrogen bonding have been omitted for clarity.

## supplementary crystallographic information

### Comment

Tetrazole derivatives have found wide range of applications in coordination chemistry because of their multiple coordination modes as ligands to metal ions and for the construction of novel metal-organic frameworks (Wang *et al.*, 2005; Xiong *et al.*, 2002). We report here the crystal structure of the title compound, 5-(2-methyl-5-nitrophenyl)-1*H*-tetrazole, (Fig.1).

The benzene ring makes a dihedral angle of 45.7 (2)° with the tetrazole ring owing to the C–C bond bridge which force the two rings to be twisted from each other. The bond distances and angles of the tetrazole rings are in the usual ranges (Wang *et al.*, 2005; Arp *et al.*, 2000; Dai & Fu, 2008).

The crystal packing is stabilized by N—H···N hydrogen bonds (Table 1), which link the molecules into chains running parallel to the *a* axis (Fig. 2). The adjacent chains are linked through π–π interactions between the tetrazole rings [centroid–centroid distance = 3.450 (2) Å] of the molecules at (*x*, *y*, *z*) and (1-*x*, 1-*y*, 1-*z*).

### Experimental

Under nitrogen protection, 2-methyl-5-nitrobenzonitrile (30 mmol), NaN_3_ (45 mmol) and NH_4_Cl (33 mmol) were added in a flask and then DMF (50 ml) was added. The mixture was stirred at 383 K for 20 h, the resulting solution was poured into ice water (100 ml) and then hydrochloric acid (6 mol/l) was added to control the pH value to 6. The white solid obtained was filtered and washed with distilled water. The crude product was recrystallized with ethanol to obtain colourless block-shaped crystals of the title compound.

### Refinement

All H atoms were positioned geometrically and treated as riding with C—H = 0.93 Å (aromatic), 0.96 Å (methyl) and N—H = 0.86 Å, and with *U*_iso_(H) = 1.2*U*_eq_(C,*N*) and *U*_iso_(H) = 1.5*U*_eq_(C_methyl_).

### Crystal data

**Table:**

C_8_H_7_N_5_O_2_	*F*(000) = 424
*M**_r_* = 205.19	*D*_x_ = 1.447 Mg m^−^^3^
Monoclinic, *P*2_1_/*c*	Mo *K*α radiation, λ = 0.71073 Å
Hall symbol: -P 2ybc	Cell parameters from 1775 reflections
*a* = 4.9057 (10) Å	θ = 2.4–27.1°
*b* = 16.938 (3) Å	µ = 0.11 mm^−^^1^
*c* = 11.463 (2) Å	*T* = 298 K
β = 98.65 (3)°	Block, colourless
*V* = 941.7 (3) Å^3^	0.25 × 0.18 × 0.15 mm
*Z* = 4	

### Data collection

**Table:**

Rigaku Mercury2 diffractometer	2085 independent reflections
Radiation source: fine-focus sealed tube	1434 reflections with *I* > 2σ(*I*)
graphite	*R*_int_ = 0.056
Detector resolution: 13.6612 pixels mm^-1^	θ_max_ = 27.2°, θ_min_ = 3.0°
ω scans	*h* = −6→6
Absorption correction: multi-scan (CrystalClear, Rigaku, 2005)	*k* = −21→21
*T*_min_ = 0.971, *T*_max_ = 0.977	*l* = −14→14
9281 measured reflections	

### Refinement

**Table:**

Refinement on *F*^2^	Primary atom site location: structure-invariant direct methods
Least-squares matrix: full	Secondary atom site location: difference Fourier map
*R*[*F*^2^ > 2σ(*F*^2^)] = 0.082	Hydrogen site location: inferred from neighbouring sites
*wR*(*F*^2^) = 0.203	H-atom parameters constrained
*S* = 1.13	*w* = 1/[σ^2^(*F*_o_^2^) + (0.0655*P*)^2^ + 0.9796*P*] where *P* = (*F*_o_^2^ + 2*F*_c_^2^)/3
2085 reflections	(Δ/σ)_max_ = 0.001
137 parameters	Δρ_max_ = 0.30 e Å^−^^3^
0 restraints	Δρ_min_ = −0.29 e Å^−^^3^

### Special details

**Table:**

Geometry. All e.s.d.'s (except the e.s.d. in the dihedral angle between two l.s. planes) are estimated using the full covariance matrix. The cell e.s.d.'s are taken into account individually in the estimation of e.s.d.'s in distances, angles and torsion angles; correlations between e.s.d.'s in cell parameters are only used when they are defined by crystal symmetry. An approximate (isotropic) treatment of cell e.s.d.'s is used for estimating e.s.d.'s involving l.s. planes.
Refinement. Refinement of *F*^2^ against ALL reflections. The weighted *R*-factor *wR* and goodness of fit *S* are based on *F*^2^, conventional *R*-factors *R* are based on *F*, with *F* set to zero for negative *F*^2^. The threshold expression of *F*^2^ > σ(*F*^2^) is used only for calculating *R*-factors(gt) *etc*. and is not relevant to the choice of reflections for refinement. *R*-factors based on *F*^2^ are statistically about twice as large as those based on *F*, and *R*- factors based on ALL data will be even larger.

### Fractional atomic coordinates and isotropic or equivalent isotropic displacement parameters (Å^2^)

**Table:**

	*x*	*y*	*z*	*U*_iso_*/*U*_eq_	
O1	−0.0317 (10)	0.86679 (19)	0.3611 (3)	0.1152 (15)	
O2	−0.2052 (8)	0.7689 (2)	0.4436 (3)	0.0930 (12)	
N1	0.5142 (5)	0.48416 (16)	0.3549 (2)	0.0425 (7)	
H1	0.6766	0.4970	0.3419	0.051*	
N4	0.0825 (5)	0.48791 (16)	0.3638 (2)	0.0416 (7)	
N3	0.1836 (6)	0.41763 (16)	0.4075 (3)	0.0473 (7)	
C1	0.2904 (6)	0.52855 (17)	0.3320 (3)	0.0360 (7)	
N2	0.4451 (6)	0.41548 (16)	0.4018 (3)	0.0470 (7)	
C2	0.2681 (6)	0.61097 (19)	0.2897 (3)	0.0397 (7)	
C7	0.3890 (7)	0.6358 (2)	0.1932 (3)	0.0485 (9)	
C3	0.1193 (7)	0.6641 (2)	0.3495 (3)	0.0426 (8)	
H3	0.0349	0.6473	0.4125	0.051*	
C4	0.1007 (7)	0.7403 (2)	0.3140 (3)	0.0506 (9)	
C6	0.3672 (8)	0.7152 (2)	0.1624 (3)	0.0565 (10)	
H6	0.4510	0.7333	0.0999	0.068*	
N5	−0.0555 (8)	0.7961 (2)	0.3775 (3)	0.0676 (10)	
C5	0.2254 (9)	0.7676 (2)	0.2220 (4)	0.0628 (11)	
H5	0.2136	0.8206	0.2006	0.075*	
C8	0.5334 (9)	0.5794 (2)	0.1215 (4)	0.0628 (11)	
H8A	0.4164	0.5347	0.0992	0.094*	
H8B	0.7020	0.5618	0.1677	0.094*	
H8C	0.5738	0.6056	0.0519	0.094*	

### Atomic displacement parameters (Å^2^)

**Table:**

	*U*^11^	*U*^22^	*U*^33^	*U*^12^	*U*^13^	*U*^23^
O1	0.197 (4)	0.0494 (19)	0.105 (3)	0.033 (2)	0.042 (3)	0.0013 (19)
O2	0.107 (3)	0.089 (2)	0.095 (3)	0.032 (2)	0.054 (2)	0.003 (2)
N1	0.0275 (13)	0.0435 (15)	0.0576 (18)	0.0007 (12)	0.0105 (12)	0.0030 (13)
N4	0.0317 (13)	0.0391 (14)	0.0558 (17)	−0.0045 (11)	0.0129 (12)	0.0006 (13)
N3	0.0422 (16)	0.0397 (15)	0.0616 (19)	−0.0060 (13)	0.0132 (14)	0.0008 (14)
C1	0.0293 (15)	0.0369 (16)	0.0428 (17)	−0.0034 (13)	0.0090 (13)	0.0002 (14)
N2	0.0434 (16)	0.0359 (14)	0.0637 (19)	0.0033 (12)	0.0144 (14)	0.0005 (13)
C2	0.0305 (15)	0.0437 (17)	0.0455 (18)	−0.0033 (13)	0.0076 (13)	0.0006 (15)
C7	0.0400 (18)	0.061 (2)	0.0447 (19)	0.0012 (16)	0.0085 (15)	0.0040 (17)
C3	0.0394 (17)	0.0451 (18)	0.0443 (19)	0.0003 (14)	0.0096 (14)	0.0048 (15)
C4	0.051 (2)	0.049 (2)	0.052 (2)	0.0093 (16)	0.0103 (17)	0.0000 (17)
C6	0.061 (2)	0.055 (2)	0.057 (2)	0.0009 (19)	0.0216 (19)	0.0211 (19)
N5	0.086 (3)	0.055 (2)	0.062 (2)	0.0235 (19)	0.013 (2)	0.0028 (18)
C5	0.070 (3)	0.050 (2)	0.071 (3)	0.008 (2)	0.019 (2)	0.016 (2)
C8	0.067 (3)	0.072 (3)	0.056 (2)	0.012 (2)	0.029 (2)	0.000 (2)

### Geometric parameters (Å, °)

**Table:**

O1—N5	1.220 (5)	C7—C8	1.505 (5)
O2—N5	1.222 (5)	C3—C4	1.352 (5)
N1—C1	1.324 (4)	C3—H3	0.93
N1—N2	1.346 (4)	C4—C5	1.377 (5)
N1—H1	0.86	C4—N5	1.477 (5)
N4—C1	1.326 (4)	C6—C5	1.371 (5)
N4—N3	1.357 (4)	C6—H6	0.93
N3—N2	1.294 (4)	C5—H5	0.93
C1—C2	1.477 (4)	C8—H8A	0.96
C2—C7	1.396 (5)	C8—H8B	0.96
C2—C3	1.401 (4)	C8—H8C	0.96
C7—C6	1.391 (5)		
			
C1—N1—N2	108.6 (2)	C3—C4—C5	122.2 (3)
C1—N1—H1	125.7	C3—C4—N5	118.7 (3)
N2—N1—H1	125.7	C5—C4—N5	119.1 (3)
C1—N4—N3	107.7 (2)	C5—C6—C7	121.7 (3)
N2—N3—N4	108.4 (2)	C5—C6—H6	119.2
N1—C1—N4	107.5 (3)	C7—C6—H6	119.2
N1—C1—C2	128.3 (3)	O1—N5—O2	123.2 (4)
N4—C1—C2	124.0 (3)	O1—N5—C4	118.9 (4)
N3—N2—N1	107.9 (3)	O2—N5—C4	117.9 (3)
C7—C2—C3	120.6 (3)	C6—C5—C4	118.8 (4)
C7—C2—C1	121.7 (3)	C6—C5—H5	120.6
C3—C2—C1	117.7 (3)	C4—C5—H5	120.6
C6—C7—C2	117.8 (3)	C7—C8—H8A	109.5
C6—C7—C8	120.0 (3)	C7—C8—H8B	109.5
C2—C7—C8	122.2 (3)	H8A—C8—H8B	109.5
C4—C3—C2	118.9 (3)	C7—C8—H8C	109.5
C4—C3—H3	120.6	H8A—C8—H8C	109.5
C2—C3—H3	120.6	H8B—C8—H8C	109.5
			
C1—N4—N3—N2	−0.4 (4)	C1—C2—C7—C8	4.1 (5)
N2—N1—C1—N4	−0.3 (4)	C7—C2—C3—C4	−1.6 (5)
N2—N1—C1—C2	174.4 (3)	C1—C2—C3—C4	178.6 (3)
N3—N4—C1—N1	0.4 (4)	C2—C3—C4—C5	−0.7 (6)
N3—N4—C1—C2	−174.5 (3)	C2—C3—C4—N5	−179.8 (3)
N4—N3—N2—N1	0.2 (4)	C2—C7—C6—C5	−1.9 (6)
C1—N1—N2—N3	0.1 (4)	C8—C7—C6—C5	176.7 (4)
N1—C1—C2—C7	49.1 (5)	C3—C4—N5—O1	166.6 (4)
N4—C1—C2—C7	−137.0 (3)	C5—C4—N5—O1	−12.5 (6)
N1—C1—C2—C3	−131.2 (3)	C3—C4—N5—O2	−14.0 (6)
N4—C1—C2—C3	42.7 (5)	C5—C4—N5—O2	166.9 (4)
C3—C2—C7—C6	2.9 (5)	C7—C6—C5—C4	−0.4 (6)
C1—C2—C7—C6	−177.4 (3)	C3—C4—C5—C6	1.7 (6)
C3—C2—C7—C8	−175.7 (3)	N5—C4—C5—C6	−179.2 (4)

### Hydrogen-bond geometry (Å, °)

**Table:**

*D*—H···*A*	*D*—H	H···*A*	*D*···*A*	*D*—H···*A*
N1—H1···N4^i^	0.86	1.98	2.775 (4)	154

Symmetry codes: (i) *x*+1, *y*, *z*.
